# Shifting Antimicrobial Susceptibility Patterns of Uropathogens: A 20‐Year Analysis of Urine Cultures

**DOI:** 10.1111/iju.70411

**Published:** 2026-03-12

**Authors:** Shinichi Takebe, Masami Endo, Nanaho Demizu, Mei Tokumoto, Ryota Morinaga, Taku Mitome, Akitoshi Takizawa

**Affiliations:** ^1^ Department of Urology International Goodwill Hospital Yokohama Kanagawa Japan; ^2^ Department of Urology Yokohama City University Hospital Yokohama Kanagawa Japan; ^3^ Department of Clinical Laboratory International Goodwill Hospital Yokohama Kanagawa Japan

**Keywords:** bacterial, beta‐lactamases, drug resistance, *Escherichia coli*, fluoroquinolones, urinary tract infections

## Abstract

**Objectives:**

Antimicrobial resistance among uropathogens, particularly extended‐spectrum β‐lactamase (ESBL) production and fluoroquinolone (FQ) resistance, presents a significant challenge to treating urinary tract infections (UTIs). This study aimed to analyze 20‐year trends in antimicrobial susceptibility among key urinary isolates at a single secondary care hospital in Japan.

**Methods:**

We retrospectively analyzed 18 873 urine culture isolates collected between January 2004 and December 2023. Bacterial identification and antimicrobial susceptibility testing were performed according to CLSI guidelines. Temporal trends in ESBL production and FQ resistance rates were analyzed using logistic regression.

**Results:**

Significant increases in ESBL production were observed in *Escherichia coli* (increasing from 1.4% to a peak of 23.6% in 2020–2021, then settling at 20.4%), *Klebsiella pneumoniae* , and *Proteus mirabilis* . FQ resistance also increased significantly in *E. coli* (from 7.8% to 33.1%), methicillin‐sensitive *Staphylococcus aureus* (MSSA), and Group B Streptococcus (GBS). Conversely, FQ resistance significantly decreased in *Enterococcus faecalis* and *Pseudomonas aeruginosa* . Although the rates of ESBL‐producing and FQ‐resistant *E. coli* increased overall, they have plateaued in recent years.

**Conclusions:**

This two‐decade surveillance revealed complex, species‐specific antimicrobial resistance patterns. The high rate of FQ resistance in *E. coli* , despite a recent plateau, underscores the importance of incorporating local susceptibility data to guide empirical antibiotic selection. Continuous, facility‐level surveillance is essential for effective antimicrobial stewardship and for optimizing patient treatment in an era of evolving resistance.

**Abbreviations**
AMR: antimicrobial resistance
AST: Antimicrobial Stewardship Team
CFU: colony‐forming unit
CLSI: Clinical and Laboratory Standards Institute
cUTI: complicated urinary tract infection
EAU: European Association of Urology
ESBL: extended‐spectrum β‐lactamase
FQ: fluoroquinolone
GBS: Group B Streptococcus
IDSA: Infectious Diseases Society of America
JAID: Japanese Association for Infectious Disease
JANIS: Japan Nosocomial Infections Surveillance
JSC: Japanese Society of Chemotherapy
LVFX: levofloxacin
MIC: minimum inhibitory concentration
MRSA: methicillin‐resistant *Staphylococcus aureus*

MSSA: methicillin‐sensitive *Staphylococcus aureus*

UTI: urinary tract infection

## 1 Introduction

Urinary tract infections (UTIs) represent a significant global health burden, affecting millions of individuals annually and placing a substantial strain on healthcare systems worldwide [1]. The management of these common infections is increasingly complicated by the global rise of antimicrobial resistance among uropathogens, a trend directly linked to patterns of antibiotic consumption [2]. Of particular concern are the increasing rates of resistance conferred by extended‐spectrum β‐lactamase (ESBL) production and resistance to fluoroquinolone (FQ), as these mechanisms compromise the effectiveness of widely used oral therapies and complicate clinical decision‐making [3].

While the overarching global trend of increasing resistance is well‐documented, significant regional variations exist, often driven by local antibiotic prescribing habits and distinct epidemiological factors [1]. This variability makes localized data analysis essential for developing effective, evidence‐based treatment strategies. Understanding local resistance profiles is not only crucial for selecting appropriate empirical antimicrobial therapy to ensure positive patient outcomes but also for implementing targeted antimicrobial stewardship programs. Therefore, long‐term, continuous surveillance at the institutional level is necessary to accurately track these dynamic temporal trends and contribute to a more complete understanding of the national resistance landscape [4].

To address this critical need for local data, this study was designed to investigate the antimicrobial susceptibility patterns of common uropathogens isolated at our institution over the past two decades. Through a retrospective analysis, we aimed to characterize the temporal changes in key resistance profiles, with a specific focus on ESBL‐producing organisms and FQ resistance. The objective is to provide crucial, region‐specific data that can be used to optimize local empirical treatment protocols and contribute valuable insights into the broader evolution of antimicrobial resistance in Japan.

## 2 Methods

This retrospective study was conducted at International Goodwill Hospital in Yokohama, Japan. The study analyzed urine culture data collected over a 20‐year period, from January 2004 to December 2023. Patients with bacteriuria (bacteria ≥ 10^4^ colony‐forming unit (CFU)/mL in midstream urine or catheterized specimens) were included in this study. To avoid overrepresentation of individual patients, duplicate urine cultures from the same patient within the same month were excluded. Bacterial identification and antimicrobial susceptibility testing were performed using the MicroScan WalkAway system (Siemens, Germany) and the broth microdilution method. The interpretation of the results followed the guidelines between 2003 and 2020 provided by the Clinical and Laboratory Standards Institute (CLSI) [5]. The antimicrobial susceptibility of the pathogens was categorized into 1 of 3 classes; namely, susceptible, intermediate, or resistant, according to the minimum inhibitory concentration (MIC) breakpoints recommended by CLSI. FQ resistance was defined as intermediate or resistant to levofloxacin (LVFX). Data collected for each urine culture included the isolated bacterial species, the antimicrobial susceptibility profile for each isolate, and the date of sample collection.

Temporal trends in antimicrobial resistance were analyzed using logistic regression, with the presence of resistance or ESBL production as the dependent variable and the calendar year as the continuous explanatory variable. The resistance rate for each antimicrobial agent was calculated biennially. Statistical analyses were performed using EZR (Saitama Medical Center, Jichi Medical University, Saitama, Japan) [6], a graphical user interface for R (The R Foundation for Statistical Computing, Vienna, Austria). A *p*‐value of < 0.05 was considered statistically significant.

Ethical approval was obtained from the Institutional Review Board of International Goodwill Hospital (approval number: 3293_07). This study conformed to the principles of the Declaration of Helsinki.

## 3 Results

A total of 18 873 urine cultures collected between January 2004 and December 2023 at International Goodwill Hospital were analyzed. Table 1 presents the distribution of bacterial species isolated from these cultures, including the total number of isolates analyzed for each biennial period. The table individually lists the eight most frequently isolated pathogens from the 2022–2023 period, with all other species consolidated into an “Others” category. Throughout the entire 20‐year study period, *Escherichia coli* was consistently the most common isolate, and *Enterococcus faecalis* was consistently the second most common. While the rankings of subsequent pathogens showed minor fluctuations across the biennial periods, the overall distribution of the primary uropathogens remained relatively stable over two decades, with no major long‐term shifts in the prevalence of any single species. In 2022–2023, the most frequently isolated species were *E. coli* (37.9%), *E. faecalis* (11.6%), *Klebsiella pneumoniae* (7.0%), and *Pseudomonas aeruginosa* (5.8%).

**TABLE 1:** Biennial distribution of bacterial species isolated from urine cultures from 2004 to 2023.

Bacterial species	2004–2005	2006–2007	2008–2009	2010–2011	2012–2013	2014–2015	2016–2017	2018–2019	2020–2021	2022–2023
	*n* (%)	*n* (%)	*n* (%)	*n* (%)	*n* (%)	*n* (%)	*n* (%)	*n* (%)	*n* (%)	*n* (%)
*E. coli*	359 (25.3)	421 (26.9)	332 (23.8)	398 (25.8)	464 (30.0)	591 (39.0)	666 (43.2)	829 (33.9)	1121 (37.8)	1113 (37.9)
*E. faecalis*	246 (17.3)	281 (18.0)	288 (20.7)	288 (18.7)	244 (15.8)	208 (13.7)	182 (11.8)	344 (14.1)	336 (11.3)	340 (11.6)
*K. pneumoniae*	60 (4.2)	58 (3.7)	67 (4.8)	60 (3.9)	95 (6.1)	105 (6.9)	135 (8.7)	171 (7.0)	214 (7.2)	205 (7.0)
*P. aeruginosa*	86 (6.1)	116 (7.4)	102 (7.3)	118 (7.6)	101 (6.5)	112 (7.4)	92 (6.0)	144 (5.9)	177 (6.0)	170 (5.8)
*S. aureus*	142 (10.0)	93 (5.9)	58 (4.2)	66 (4.3)	78 (5.0)	61 (4.0)	76 (4.9)	114 (4.7)	132 (4.5)	125 (4.3)
MSSA	74 (5.2)	58 (3.7)	36 (2.6)	39 (2.5)	46 (3.0)	39 (2.6)	36 (2.3)	78 (3.2)	77 (2.6)	92 (3.1)
MRSA	68 (4.8)	35 (2.2)	22 (1.6)	27 (1.7)	32 (2.1)	22 (1.5)	40 (2.6)	36 (1.5)	55 (1.9)	33 (1.1)
GBS	104 (7.3)	78 (5.0)	64 (4.6)	88 (5.7)	71 (4.6)	34 (2.2)	32 (2.1)	91 (3.7)	138 (4.7)	114 (3.9)
*P. mirabilis*	10 (0.7)	29 (1.9)	31 (2.2)	46 (3.0)	25 (1.6)	39 (2.6)	50 (3.2)	77 (3.1)	91 (3.1)	89 (3.0)
*K. oxytoca*	12 (0.8)	18 (1.2)	22 (1.6)	30 (1.9)	31 (2.0)	30 (2.0)	21 (1.4)	46 (1.9)	40 (1.3)	60 (2.0)
Others	401 (28.2)	470 (30.1)	429 (30.8)	449 (29.1)	440 (28.4)	334 (22.1)	289 (18.7)	629 (25.7)	717 (24.2)	720 (24.5)
Total	1420 (100.0)	1564 (100.0)	1393 (100.0)	1543 (100.0)	1549 (100.0)	1514 (100.0)	1543 (100.0)	2445 (100.0)	2966 (100.0)	2936 (100.0)

Abbreviations: GBS, Group B Streptococcus; MRSA, methicillin‐resistant *Staphylococcus aureus* ; MSSA, methicillin‐sensitive *Staphylococcus aureus* .

### 3.1 ESBL Production Trends

**FIGURE 1:**
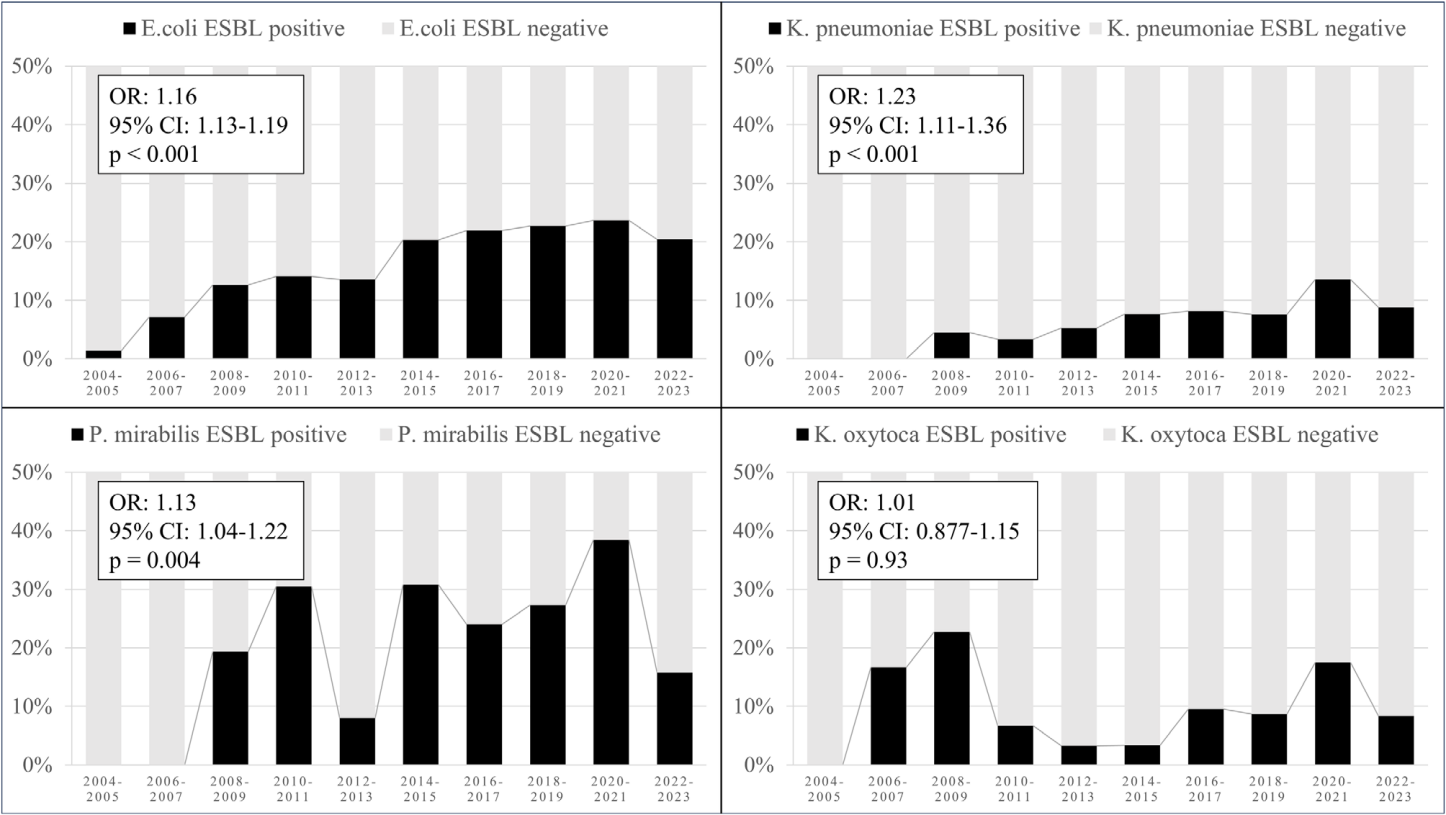
Trends in Extended‐spectrum β‐lactamase (ESBL) production in *E. coli* , *K. pneumoniae* , *P. mirabilis* , and *K. oxytoca* from 2004 to 2023. Logistic regression analysis results (Odds ratio [OR], 95% Confidence Interval [CI] and *p*‐value) are displayed within each panel.

The biennial trends in ESBL production among *E. coli* , *K. pneumoniae* , *Proteus mirabilis* , and *Klebsiella oxytoca* , including logistic regression analysis results, are shown in Figure 1. The proportion of ESBL‐producing *E. coli* significantly increased, rising from approximately 1.4% in 2004–2005 to 23.6% in 2020–2021, but this trend has plateaued, with a rate of 20.4% in 2022–2023. Consistent with the trend observed in *E. coli* , both *K. pneumoniae* and *P. mirabilis* exhibited significantly increasing ESBL‐producing rates. The analysis of *K. oxytoca* was limited by the low number of isolates, resulting in high variability and a lack of statistical significance in the observed trend.

### 3.2 FQ Resistance Trends

**FIGURE 2:**
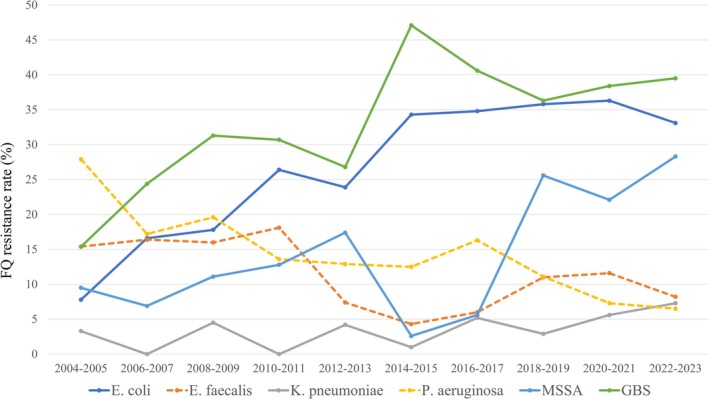
Trends in fluoroquinolone (FQ) resistance rates among frequent bacterial species from 2004 to 2023. Statistically significant increases are indicated by solid lines and decreases are indicated by dotted lines.

Table 2 shows biennial trends in FQ resistance of frequent bacterial species, including isolate counts, percentages of resistant strains, and logistic regression analysis results. Statistically significant increases in FQ resistance were observed for *E. coli* , *K. pneumoniae* , Methicillin‐sensitive *Staphylococcus aureus* (MSSA), and Group B Streptococcus (GBS). FQ resistance of *E. coli* increased steadily from 7.8% in 2004–2005 to 34.3% in 2014–2015; however, it has plateaued in the years since. Conversely, statistically significant decreases were observed for *E. faecalis* and *P. aeruginosa* (Figure 2). The trends for Methicillin‐resistant *Staphylococcus aureus* (MRSA) and overall *S. aureus* were not statistically significant.

**TABLE 2:** Biennial distribution of fluoroquinolone (FQ) resistance of frequent bacterial species from 2004 to 2023.

Bacterial species	Number of FQ resistant strains										Odds ratio (95% CI)	*p*
	2004–2005	2006–2007	2008–2009	2010–2011	2012–2013	2014–2015	2016–2017	2018–2019	2020–2021	2022–2023		
	*n* (%)	*n* (%)	*n* (%)	*n* (%)	*n* (%)	*n* (%)	*n* (%)	*n* (%)	*n* (%)	*n* (%)		
*E. coli*	28 (7.8)	70 (16.6)	59 (17.8)	105 (26.4)	111 (23.9)	203 (34.3)	232 (34.8)	297 (35.8)	407 (36.3)	368 (33.1)	1.14 (1.12–1.17)	< 0.001
*E. faecalis*	38 (15.4)	46 (16.4)	46 (16.0)	52 (18.1)	18 (7.4)	9 (4.3)	11 (6.0)	38 (11.0)	39 (11.6)	28 (8.2)	0.915 (0.88–0.952)	< 0.001
*K. pneumoniae*	2 (3.3)	0 (0.0)	3 (4.5)	0 (0.0)	4 (4.2)	1 (1.0)	7 (5.2)	5 (2.9)	12 (5.6)	15 (7.3)	1.18 (1.04–1.34)	0.010
*P. aeruginosa*	24 (27.9)	20 (17.2)	20 (19.6)	16 (13.6)	13 (12.9)	14 (12.5)	15 (16.3)	16 (11.1)	13 (7.3)	11 (6.5)	0.86 (0.812–0.911)	< 0.001
*S. aureus*	68 (47.9)	37 (39.8)	25 (43.1)	30 (45.5)	37 (47.4)	22 (36.1)	41 (53.9)	55 (48.2)	71 (53.8)	54 (43.2)	1.02 (0.978–1.06)	0.37
MSSA	7 (9.5)	4 (6.9)	4 (11.1)	5 (12.8)	8 (17.4)	1 (2.6)	2 (5.6)	20 (25.6)	17 (22.1)	26 (28.3)	1.18 (1.1–1.28)	< 0.001
MRSA	61 (89.7)	33 (94.3)	21 (95.5)	25 (92.6)	29 (90.6)	21 (95.5)	39 (97.5)	35 (97.2)	54 (98.2)	28 (84.8)	1.06 (0.927–1.21)	0.40
GBS	16 (15.4)	19 (24.4)	20 (31.3)	27 (30.7)	19 (26.8)	16 (47.1)	13 (40.6)	33 (36.3)	53 (38.4)	45 (39.5)	1.11 (1.06–1.17)	< 0.001
*P. mirabilis*	0 (0.0)	2 (6.9)	3 (9.7)	11 (23.9)	2 (8.0)	14 (35.9)	10 (20.0)	18 (23.4)	20 (22.0)	11 (12.4)	1.05 (0.959–1.15)	0.30
*K. oxytoca*	0 (0.0)	3 (16.7)	5 (22.7)	1 (3.3)	1 (3.2)	0 (0.0)	2 (9.5)	1 (2.2)	1 (2.5)	3 (5.0)	0.846 (0.71–1.01)	0.061

Abbreviations: CI, confidence interval; FQ, fluoroquinolone; GBS, Group B Streptococcus; MRSA, methicillin‐resistant *Staphylococcus aureus* ; MSSA, methicillin‐sensitive *Staphylococcus aureus* .

## 4 Discussion

In uncomplicated cystitis, *E. coli* is reported to account for approximately 70% of causative pathogens [3]. In contrast, the proportion of *E. coli* decreases in complicated urinary tract infections (cUTIs), while the prevalence of species such as *E. faecalis* and *P. aeruginosa* increases [7, 8]. The distribution of urinary isolates in this study closely resembled the pathogen distribution reported for cUTIs. This is likely attributable to our institution's role as a secondary care hospital, where the majority of treated UTIs are complicated in nature. Although the major bacterial species have not changed significantly over the study period, we observed a gradual shift in the composition of isolates: the proportion of Gram‐negative rods, including *E. coli* and *K. pneumoniae* , has increased, while Gram‐positive bacteria such as *E. faecalis* have decreased. This trend may be partly attributed to the rapid increase in ESBL‐producing strains among Gram‐negative bacteria. This 20‐year surveillance study provides a comprehensive analysis of antimicrobial susceptibility trends among these uropathogens, revealing two key findings. First, we observed a significant increase in ESBL production among *E. coli* , *K. pneumoniae* , and *P. mirabilis* isolates, although the rate of increase for *E. coli* appears to have plateaued in recent years. Second, while FQ resistance increased significantly in *E. coli* , *K. pneumoniae* , MSSA, and GBS, a significant decreasing trend was observed in *E. faecalis* , *P. aeruginosa* , and *K. oxytoca* . These findings highlight the dynamic nature of antimicrobial resistance and underscore the importance of ongoing local surveillance to inform appropriate treatment strategies.

The increasing prevalence of ESBL‐producing bacteria observed in our study is consistent with trends reported in Japanese nationwide surveillance programs for cUTIs. These national reports documented a progressive rise in ESBL‐producing *E. coli* , with rates increasing from 5.1% in 2008 to 15.2% in 2011, 24.3% in 2015, and reaching 24.8% by 2020–2021 [7, 9, 10, 11]. Similarly, the national rate for ESBL‐producing *K. pneumoniae* increased from 0% in 2008 to 4.5% in 2011, 7.7% in 2015, and 16.2% in 2020–2021. An upward trend was also noted for *P. mirabilis* , whereas ESBL‐producing strains were rarely detected among *K. oxytoca* isolates. A similar, though less pronounced, increase in ESBL‐producing *E. coli* has been documented in uncomplicated UTIs, rising from 4.7% in 2009 to 10.6% in 2020 [3, 4, 12, 13]. In our study, although the proportion of ESBL‐producing *E. coli* had been increasing, it peaked around 2020 and has since shown a slight decreasing trend. This favorable change might be attributed to the achievements of the Antimicrobial Stewardship Team (AST) within our hospital and regional efforts to promote appropriate antimicrobial use, in alignment with the National Action Plan on Antimicrobial Resistance (AMR) established by the Japanese Ministry of Health, Labour and Welfare [14]. This pattern is not unique to Japan, as numerous studies worldwide have documented the rising prevalence of ESBL‐producing *E. coli* and *K. pneumoniae* in UTIs [1, 15, 16, 17, 18, 19, 20, 21]. However, direct comparison of results, particularly regarding temporal trends in resistance rates, is often challenging due to differences in study design and surveillance methodologies [22]. A key strength of the present study is its single‐center design with a standardized methodology, which likely provides an accurate reflection of local ESBL trends over the past two decades.

FQ resistance in *E. coli* is a well‐documented concern, not only in Japan but also in other parts of the world [23, 24, 25]. In Japan, nationwide surveillance of cUTIs revealed that 44.5% of *E. coli* isolates were resistant to LVFX in 2020–2021, representing a continuous increase from 28.6% in 2008, 29.6% in 2011, and 38.5% in 2015 [7, 9, 10, 11]. Although FQ resistance rates in *E. coli* from uncomplicated UTIs are lower, they also showed an upward trend, increasing from 13.3% in 2015 to 18.6% in 2020. Resistance is particularly high among postmenopausal women, where 32.1% of *E. coli* isolates were FQ‐resistant in 2020 [3, 4, 13]. A separate 30‐year surveillance study from a single university hospital in Japan reported that while the isolation rates of *P. aeruginosa* and MRSA from cUTIs have decreased, there has been a corresponding relative increase in the isolation of FQ‐resistant *E. coli* . In that study, the FQ resistance rate for *E. coli* was approximately 35% in 2013, a rate comparable to that observed in our study during the same period [26]. According to the National Action Plan on Antimicrobial Resistance (AMR) 2023–2027 [14], the FQ resistance rate of *E. coli* in Japan was reported to be 41.5% in 2020, with a target of reducing it to 30% or less by 2027. The results at our hospital indicate that resistance rates are already trending close to this target figure.

When interpreting these trends in comparison with the Japan Nosocomial Infections Surveillance (JANIS) [27], caution is required regarding data sources. A limitation of JANIS is that it aggregates isolates from all specimen sources (e.g., respiratory, blood, urine), whereas resistance patterns can vary by infection site due to differences in antibiotic penetration and selection pressures. A major strength of our study is its exclusive focus on urine cultures, providing specific antibiograms directly applicable to UTI management. Notably, the increasing trend of resistance in *E. coli* observed in our study parallels the national trends reported by JANIS [27]. This similarity suggests that the increasing resistance trend reported in national surveillance, which encompasses various specimen types, is consistent with the trend specifically observed in urinary isolates. Furthermore, our consistent 20‐year single‐center methodology eliminates the bias of changing facility participation that can affect national surveillance data, offering a robust validation of these long‐term trends.

These findings have significant implications for the clinical management of UTIs in Japan. The 2023 guidelines from the Japanese Association for Infectious Disease (JAID) and the Japanese Society of Chemotherapy (JSC) recommend clavulanic acid/amoxicillin, sulbenicillin, LVFX, ciprofloxacin, tosufloxacin, and sitafloxacin as first‐line agents for complicated cystitis [28]. In contrast, guidelines from the European Association of Urology (EAU) and the Infectious Diseases Society of America (IDSA) recommend that FQ should only be prescribed when other antibiotics are inappropriate, and their use should only be considered when local FQ resistance rates are below 10% [29]. Considering the high and increasing FQ resistance rate in *E. coli* documented in our region and nationwide, the empirical selection of FQ may be an inappropriate strategy. Evidence suggests that modifying prescribing habits can successfully lower resistance; a study in the United States demonstrated that after guideline changes reduced FQ prescribing for acute uncomplicated cystitis, FQ resistance in *E. coli* significantly decreased [30]. Notably, our study observed a significant decrease in FQ resistance among *E. faecalis* , *P. aeruginosa* , and *K. oxytoca* . Reducing the resistance rate to FQ is particularly critical for *P. aeruginosa* , as this class represents the only orally available antimicrobial option with activity against this pathogen. In our study, the resistance rate of *P. aeruginosa* progressively decreased from 27.9% in 2004–2005 to 6.5% in 2022–2023. This improvement likely reflects the effectiveness of hospital‐wide efforts led by the AST to ensure appropriate antimicrobial use and avoid the casual prescription of FQ. To maintain this favorable trend and preserve the efficacy of FQ against these pathogens, the selective use of this antibiotic class must be encouraged.

This study has several limitations. First, as a single‐center, retrospective analysis, the results are specific to our region and may not be generalizable to all of Japan. Second, our data collection was limited to the MicroScan WalkAway system, which is not linked to the electronic medical record system at our institution. Therefore, we could not extract clinical information such as patient age, sex, underlying diseases, or clinical symptoms, limiting our analysis to urine culture results alone. Consequently, we were unable to differentiate between uncomplicated and complicated UTIs, cystitis and pyelonephritis, or symptomatic infections and asymptomatic bacteriuria. Third, our analysis was based only on positive urine cultures, and we could not determine the total number of cultures submitted or the overall positivity rate. These limitations suggest that a larger, continuous surveillance program is necessary to correct for regional biases and to incorporate clinical data for a more detailed analysis.

In conclusion, this two‐decade surveillance revealed complex antimicrobial resistance patterns, characterized by significant increases in both ESBL production and FQ resistance in key uropathogens like *E. coli* , alongside decreasing FQ resistance in other species such as *E. faecalis* and *P. aeruginosa* . The high rate of FQ resistance in *E. coli* , although recently plateaued, highlights the importance of incorporating local susceptibility data to inform empirical antibiotic selection as recommended by guidelines. These findings demonstrate that continuous local surveillance is essential for effective antimicrobial stewardship and for guiding evidence‐based prescribing practices in an era of evolving resistance.

## Author Contributions

**Shinichi Takebe:** conceptualization, writing – original draft, writing – review and editing, investigation, formal analysis, data curation. **Masami Endo:** data curation. **Nanaho Demizu:** data curation. **Mei Tokumoto:** data curation. **Ryota Morinaga:** data curation. **Taku Mitome:** data curation. **Akitoshi Takizawa:** project administration.

## Ethics Statement

The protocol for this research project has been approved by a suitably constituted Ethics Committee of the institution and it conforms to the provisions of the Declaration of Helsinki. Institutional Review Board of International Goodwill Hospital, Approval No. 3293_07.

## Consent

This study was a retrospective study; we applied the opt‐out method to obtain consent on this study.

## Conflicts of Interest

The authors declare no conflicts of interest.
